# Macrosight: A Novel Framework to Analyze the Shape and Movement of Interacting Macrophages Using Matlab^®^ †

**DOI:** 10.3390/jimaging5010017

**Published:** 2019-01-14

**Authors:** José Alonso Solís-Lemus, Brian Stramer, Greg Slabaugh, Constantino Carlos Reyes-Aldasoro

**Affiliations:** 1 School of Mathematics, Computer Science and Engineering, City, University of London, London EC1V 0HB, UK; 2 Randall Division of Cell & Molecular Biophysics, King’s College London, London WC2R 2LS, UK

**Keywords:** segmentation, macrophages, overlapping objects, shape analysis, movement analysis

## Abstract

This paper presents a novel software framework, called macrosight, which incorporates routines to detect, track, and analyze the shape and movement of objects, with special emphasis on macrophages. The key feature presented in macrosight consists of an algorithm to assess the changes of direction derived from cell–cell contact, where an interaction is assumed to occur. The main biological motivation is the determination of certain cell interactions influencing cell migration. Thus, the main objective of this work is to provide insights into the notion that interactions between cell structures cause a change in orientation. Macrosight analyzes the change of direction of cells before and after they come in contact with another cell. Interactions are determined when the cells overlap and form *clumps* of two or more cells. The framework integrates a segmentation technique capable of detecting overlapping cells and a tracking framework into a tool for the analysis of the trajectories of cells before and after they overlap. Preliminary results show promise into the analysis and the hypothesis proposed, and lays the groundwork for further developments. The extensive experimentation and data analysis show, with statistical significance, that under certain conditions, the movement changes before and after an interaction are different from movement in controlled cases.

## 1. Introduction

The migration of cells is of great importance in several biological processes, such as embryogenesis, wound healing and, more relevantly, the immune system [1,2,3]. Macrophages are cells of the immune system that filter foreign particles when settled in lymphoid tissues and the liver [1]. In homeostasis, or the tendency to an equilibrium of physiological processes, the role of macrophages ranges from tissue repair through to immune responses to pathogens [4]. However, excessive migration can be related to autoimmune diseases and cancer [5]. The common fruit fly, *Drosophila melanogaster*, has been widely studied as a model organism on developmental and cellular processes relating to other organisms, including humans [6]; such investigations have led to insights into how macrophages integrate external cues into migration [2]. For example, in [7,8], previously unrevealed dynamics of cytoskeletal structures in macrophages were discovered; where certain events of cell–cell contact appeared to anticipate migration.

Cell tracking is defined as the linking between detected objects in one time frame to the objects in subsequent frames. In this work, tracking is defined as a function of segmentation, the correct identification of cells from the background and from each other. Both cell segmentation and tracking have been widely studied [9,10,11,12]. Cell-tracking studies of phase-contrast microscopy time sequences have been presented in [9,10], showing quantitative analysis of cell dynamics in vitro. In [11,12], several tracking algorithms were evaluated with a series migratory cells with very different conditions, not only in their ability to track detected objects, but also to identify important cellular events, like mitosis. Other cellular events, such as the interactions between cells are also of importance. To assess such events, a more thorough study of a tracks’ features is required.

The analysis of movement, defined as the analysis of track features, is performed to answer a specific research question about the phenomenon studied. For example, in [13], tracks are classified based on their features such as speed and curvature. In a related implementation, an automated analysis of movement patterns provided a toxicological assessment of the environment [14]. In this assessment, the tracks produced by the movement of marine crustaceans were examined to detect specific patterns of motion indicating levels of toxicity in the environment. Finally, contributions regarding the specific data analyzed in this work have been varied. Segmentation of macrophages in single frames was presented in [15], showcasing the complex interactions which manifest as overlapping (*clumps*). In [16], the relationship between contiguous frames was incorporated to the segmentation of single cells, allowing for a controlled measurement of shape parameters between overlapping events.

In this work, a software framework to analyze the movement and the changes of shape of fluorescently labelled macrophages is presented. The framework, called macrosight, consists of two stages. The first stage segments and tracks the cells [15,17]. The second stage, contains two types of analyses from the tracking results: (a) the shape tracking described in [16] and (b) the analysis of interactions introduced in [18]. In the latter, overlapped *clumps* are considered moments of assumed interaction between the cells and thus the movement before and after contact is analyzed.

**Figure 1:**
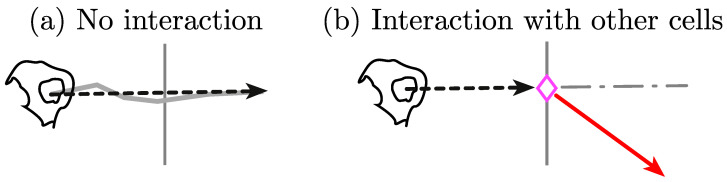
Illustration of the main hypothesis in this work. The interactions of the cells in a *clump* appear to influence on the migration patterns of the cells. The diagram shows (**a**) the case where a cell’s trajectory does not change significantly from a point chosen arbitrarily, where the grey line segments correspond to the trajectory of the cell; (**b**) shows the expectation of a cell that interacting in a *clump* and changing direction noticeably, the marker (⋄, magenta) represents a cell-cell contact. In (**b**), dotted arrows represent the direction of the cell, before (black) and after (red) contact.

The two main hypotheses of this work are (i) that cell–cell contact accounts for an interaction between cells, and (ii) as a result of an interaction, one or both cells involved in the interaction will noticeably change the direction before and after contact. Figure 1 shows a graphical abstract of the main contribution of the direction changing in the algorithm.

A preliminary version of this work was presented at the 22nd Medical Image Understanding and Analysis (MIUA) [18]. The algorithms have been extended and several new experiments with new data are presented. Thus, this work now describes the following topics, not included previously: (i) description of the macrosight framework, a complete framework for the analysis of movement of macrophages; (ii) a thorough descriptive statistical analysis of the cell–cell interactions, providing insights on interactions occurring after cell contact; (iii) more experiments performed on two additional datasets, comprising an increment of the sample size from 20 to over 50 cases analyzed.

## 2. Materials

Three different experiments of fluorescently labelled macrophages of the model organism *Drosophila melanogaster* were observed, producing three distinct videos of migrating macrophages. In each of the experiments, the nuclei were labelled with CFP-Moesin, while the microtubules of the cells were labelled with Clip-GFP, a microtubule probe; the complete imaging methodology is thoroughly described in [7,8]. The images were obtained at a pixel density of 0.21 μm/pixel and every 10 s. From the three datasets, RGB images were created by putting the nuclei into red layer and the microtubules into the green layer, resulting in a size of $\left(n_{w},n_{h},n_{d}\right)=\left(512,672,3\right)$ and two layers of fluorescence. For a simpler identification, the datasets will be named as MACROS1, MACROS2 or MACROS3, where the number of frames per dataset is 541, 361 and 462 frames, respectively.

**Figure 2:**
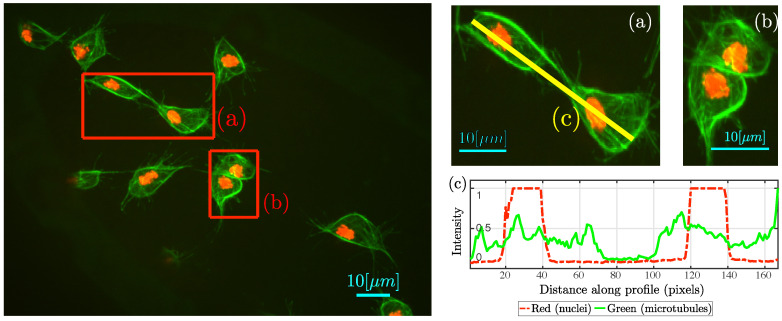
Representative time frame highlighting in red (**a**,**b**) the interactions between cells; (**c**) Shows the intensities for the red and green channel along an image profile (yellow, solid). Detail of the highlighted pairs of cells, (**a**,**b**) are shown. (**a**) Notice the arm-like microtubular structures in green, identified in [8] as moments before a change in trajectories. A bigger portion of the cells are in contact in (**b**), showing and example where cell–cell contact occurs over several frames. Bars 10 μm.

**Figure 3:**
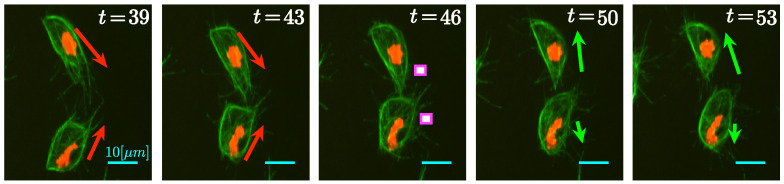
Representation of the movement of two cells before and after the interaction (*clump*). Five frames are shown with arrows which represent the observed direction of movement, before (red) and after (green) the *clump*. The sizes of the arrows represent the observed speed of movement. Frame 46, in the center, shows an interaction between the two moving cells, forming a *clump*. Bars 10 μm.

Overlapping events, referred to as *clumps*, are relevant to the study of the datasets. Figure 2 contains an example frame and detail of *clumps* where cell–cell contact can be appreciated in two distinct ways: Figure 2a shows a pair of cells where microtubular structures align; Figure 2b shows two cells completely overlapped. Empirical observation of the data in [8] suggest that cell–cell contact, similar to the one showed in Figure 2a, could influence the migration pattern of the cells involved, with interactions considered to be under 3 min (18 frames). For example, the cells shown in Figure 3, where a series of frames is shown, taken from two of cells that overlap and then appear to change direction over 15 frames.

## 3. Methods

In this section, the main functionalities of macrosight, in the context of direction changes will be presented. Special emphasis will be given to the algorithms analyzing changes of cell direction as a result of cell–cell contact, which will be the focus of this work. In a previous development [15], *clumps* were studied and how to separate them. In this work, *clumps* will be considered points of interaction between cells. In Figure 3, arrows indicate the observed direction of movement of the cells. Notice the green microtubules aligning in the top cell on frames t=39 and t=43, just before and during the contact between cells. Such alignment has been reported [8] to indicate the change of trajectories between the cells in the following frames.

**Figure 4:**
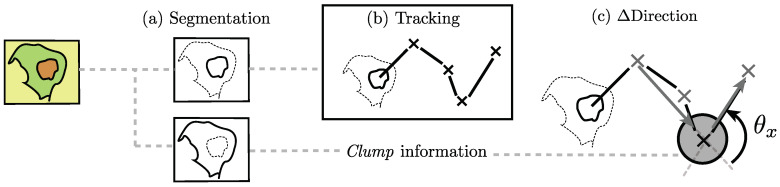
Graphical illustration of the analysis done in this work. Three principal phases are depicted. (**a**) Segmentation of each fluorescence channel; (**b**) Tracking of the red channel and identification of each *clump*. Each mark (×) in the diagram corresponds to a different time frame; (**c**) Finally, the measuring of the change of direction angle ($\theta_{x}$) before and after a detected *clump*.

The methodology for cell–cell contact influence in the change of direction can be divided into four stages of analysis. First, the segmentation of each channel individually. Then, the tracking of the objects detected in the red channel is performed, and the detections of each channel are classified as *clumps* or single cells. Finally, for each track, the change of direction (Δ Direction) is found before and after a given *clump*. Figure 4 shows a graphical representation of the procedures carried out in this work. Each stage is detailed in the following sections.

### 3.1. Segmentation of Fluorescence Intensity Channels

The segmentation procedure for segmenting each of the respective channels follows three steps, fully described in [15]. Following a low-pass filtering, each channel was segmented by a hysteresis thresholding technique [17]. Finally, a morphological opening with a disk structural element (r=3) was performed to remove noise and smooth the edges. Detection of *clumps* was achieved by comparing the number of nuclei detected within the area covered by each segmentation of the green channel.

### 3.2. Tracking of the Nuclei and Incorporation of *Clump* Information

Tracking is defined as the linking of detected objects between consecutive time frames, namely: parent (t-1), present (*t*) and child (t+1). Following segmentation, the tracking of the objects in the RED channel was performed through the PhagoSight software [17,19], a framework for cell tracking that uses the Keyhole algorithm [20,21]. The macrosight framework incorporates the segmentation of the RED channel to PhagoSight, for tracking, and incorporates the information of the *clumps* in the GREEN channel after the tracks have been calculated.

**The Keyhole Tracking Algorithm.** The algorithm [17,20] links the segmented objects in contiguous frames through the analysis of the velocity and direction of the object at frames t-1 and *t*, and estimates the position of the object at frame t+1. Apart from the estimation, the algorithm generates two regions of probability, which resemble an old-style keyhole, to anticipate changes in trajectory. Each track produced by PhagoSight includes the information of the cell’s nuclei that has been tracked and linked from one frame to others.

**Addition of *clump* information by**macrosight. The information of interest to this work is explained in Table 1, it includes parameters such as time frame, position, and velocity of each nuclei at each time frame. At each point in time, the presence of *clumps* was detected by counting the number of nuclei contained within a single object detected in the green channel. A new parameter, called clump code, has been incorporated to Table 1. Each nucleus within a *clump* has a track associated with them, thus each *clump* can be uniquely identified via a simple unique identifier number or for short *clump* code, which includes the labels of the tracks contained within it. For instance, let r,q be the labels of two tracks (r<q) which at a certain point in time belong to a *clump*, then the code *c* is defined by c(r,q)=c(q,r)=1000q+r. The value of 1000 is chosen arbitrarily as a large number, larger than the total number of tracks. Notice how the tracks’ labels are arranged from left to right starting with the highest identifier to the lowest; for example, code 24013 would correspond to a *clump* that at a certain frame contains tracks 24 and 13.

The previous definition can be extended for an arbitrary number of labels *m* interacting in the same *clump* as $c\left(r_{1},\cdots ,r_{i},\cdots ,r_{m}\right)=\sum_{i=1}^{m}1000^{i-1}r_{i}$, where all labels in the *clump* are ordered $r_{1}<\cdots <r_{i}<r_{m}$. Each *clump* can be uniquely identified based on the tracks contained in it. Table 2 shows a simple example of the creation of the *clump* codes. The inclusion of the codes facilitates the analysis of the cells that interact with each other. four cells

**Figure 5:**
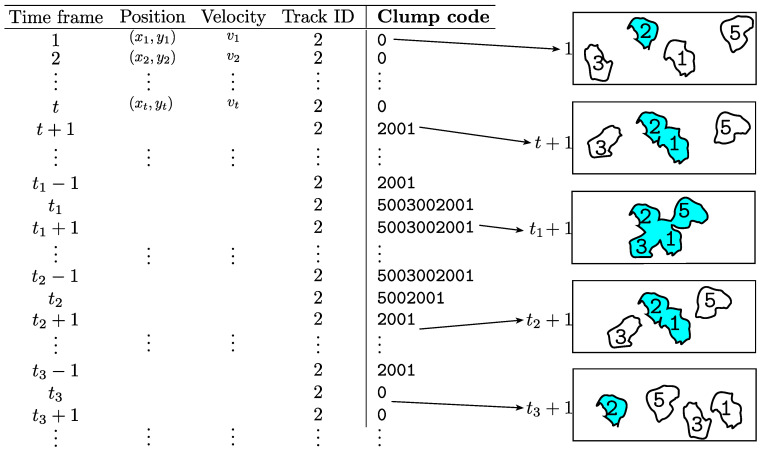
Illustration of *clump* codes incorporated to a particular track information. The table shows the information of track 2 spanning along certain time frames and in several *clumps*. The right column shows a representation of the cells at different time frames, and their involvement in different *clumps*, highlighted in blue.

Figure 5 represents the information from each track, as produced by PhagoSight, where the *clump* code is incorporated by macrosight. The information of a single track (track ID = 2) is shown, at different time frames. When the cell is not part of a *clump*, the variable *clump* code has a 0. At a specific frame t+1, the cells in the green channel come in contact, forming *clump* 2001. At frame $t_{1}$, two more cells come in contact with *clump* 2001, thus forming *clump* 5003002001.

### 3.3. Measuring the Change of Direction before and after a *Clump*

The algorithm developed in this work, estimates the angle formed between the direction of the cell prior to an interaction (*clump*) and the direction of the cell once the interaction is over and it does not belong to any *clump*. Let $\theta_{x}\in \left(-\pi ,\pi \right)$ be the angle that measures the direction change (Δ direction). Let a track with label *r*, given by $T_{r}=\left\{\left(x_{t},y_{t}\right)\in R^{2}|t=t_{1},\cdots ,t_{T}\right\}$, interact with another $T_{q}$ through a *clump* with code c(r,q), such that the overlap between the two cells happens at time frames $t_{k_{0}},t_{k_{1}},\cdots ,t_{k_{C}}$. Let $\theta_{x}\in \left(-\pi ,\pi \right)$ be the angle that measures the direction change (Δ direction). Let a track with label *r*, given by $T_{r}=\left\{\left(x_{t},y_{t}\right)\in R^{2}|t=t_{1},\cdots ,t_{T}\right\}$, interact with another $T_{q}$ through a *clump* with code c(r,q), such that the overlap between the two cells happens at time frames $t_{k_{0}},t_{k_{1}},\cdots ,t_{k_{C}}$.

**Figure 6:**
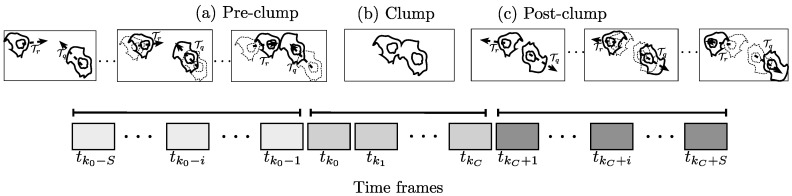
Diagram explaining the time frames chosen from tracks $T_{r},T_{q}$ for the analysis of direction change. The time frames chosen for the analysis are *S* frames before $t_{k_{0}}$ and *S* frames after $t_{k_{C}}$. The time frames are selected, and schematics of the cells moving are shown for each stage.

The determination of $\theta_{x}$ involves analyzing the tracks $T_{r},T_{q}$ starting *S* frames before $t_{k_{0}}$ and finalizing at *S* frames after $t_{k_{C}}$. Frames $t_{k_{0}-S},\cdots ,t_{k_{C}+S}$ will be referred to as the *clump span*; likewise, the time frames where the tracks are interacting, $t_{k_{0}},t_{k_{1}},\cdots ,t_{k_{C}}$, will be referred to as *time in clump*. Figure 6 shows a schematic of the tracks analyzed and the choice of the time frames. The relationship between the frames $t_{k_{0}}$, *S* and $T_{k_{C}}$ can be clearly observed as the moments in the *clump span* containing tracks $T_{r}$ and $T_{q}$. The moments are called (a) pre-*clump*, (b) *clump* and (c) post-*clump*.

**Figure 7:**
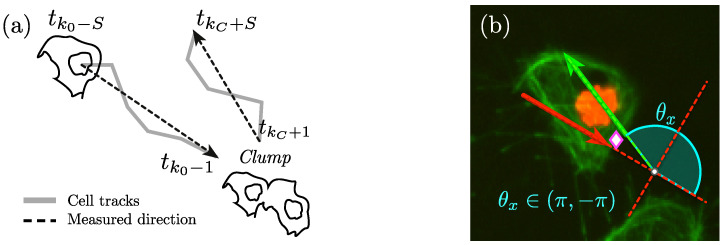
Illustration of the calculation of the angle of direction change ($\theta_{x}$). (**a**) Shows the determination of the direction before and after the *clump*; while (**b**) displays the calculation of the angle $\theta_{x}$ (cyan) from the previously selected lines. The red arrow represents the line generated by points at times $t_{k_{0}-S}$ and $t_{k_{0}-1}$, while the green arrow shows the points at times $t_{k_{C}+1}$ and $t_{k_{C}+S}$.

Once the tracks involved, and the span have been manually selected, the calculation of the change of direction angle is done by selecting a vector oriented towards the *clump* and another one leaving it. Figure 7 displays the process of selecting the lines from which direction before and after the *clump* will be selected, and the way the angle will be measured. The estimation assesses the change of direction of each cell that exits a *clump*, relative to the orientation it has entering it. To calculate the angle, vectors must be aligned and rotated from the original positions in the image (x,y) to a new set of rotated axes $\left(x^{\prime },y^{\prime }\right)$. This is performed in an intermediate step, where the incidence angle is calculated and all the points in the track are rotated to the new axes.

**Figure 8:**
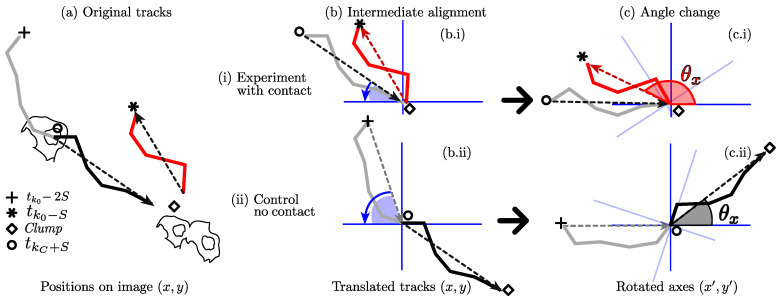
Measurement of angle change with cell–cell contact (i) and control (ii) experiments. Four markers are highlighted corresponding to specific time frames in each experiment. In order, the markers are: (+) 2S frames before contact; (∘) *S* frames before contact; (⋄) starting instant of the *clump*; and (*) *S* frames after the *clump* has finished. (**a**) Shows the original image with their original positions (x,y); (**b**) Shows the intermediate rotation of the tracks, where the tracks are rotated onto a common axis; (**c**) Represents the actual calculation of $\theta_{x}$. Trajectories show colours representing the portions of the tracks compared in contact (red) and control (black) experiments.

Figure 8 represents the calculation of the angles compared for the interactions and the control experiments. Notice that the new axes $\left(x^{\prime },y^{\prime }\right)$ in Figure 8b can be interpreted as a new frame of reference, containing all the positions rotated and aligned.

### 3.4. Experiments

All three datasets were segmented in both and tracked. The tracks’ information was searched to find cases of *clumps* that fulfil the following criteria:

1. **Only two cells interacting.** There are cases where more than one cell integrates a *clump*. These cases were excluded from the analysis as it is not clear whether the interaction of more than two cells would be different from the interaction between a pair.
2. **In and out cases.** The cases selected only involved cells with a well-defined *clump span*, in which the cell would enter the *clump* and exit it without disappearing or interacting with other *clumps*.
3. **Immediate reaction.** A small value for S=5 was chosen to define the *clump span*, as the interest of this paper is to study the immediate reaction of a cell after interacting within a *clump*.
4. **Both cells in *clump*.** Cases where both tracks in the *clump* had a well-defined *clump span* were preferred as they would allow an analysis per *clump*.

Once the tracks were selected, the $\theta_{x}$ angles were calculated for each case. Additionally, control movements for each track, consisting of the 2S time frames leading up to the *clump*, were selected to allow a comparison of the change of direction with a cell that has not interacted with another one.

The experiments were chosen in a semi-automatic way, using the information from the table in Figure 5 to generate candidates of experiments and manually logging the starting and ending points $t_{k_{0}}-2S,t_{k_{0}}-S,t_{k_{0}}$ and $t_{k_{C}}+S$ per experiment. It is important to notice that the experiments were chosen without considering the *time in clump*. As highlighted in Section 2, such parameter could be influential to the results, as the window where the interaction is observed occurs within a few minutes, which would be translated to a cell belonging a small number of time frames in *clump*.

## 4. Results

**Figure 9:**
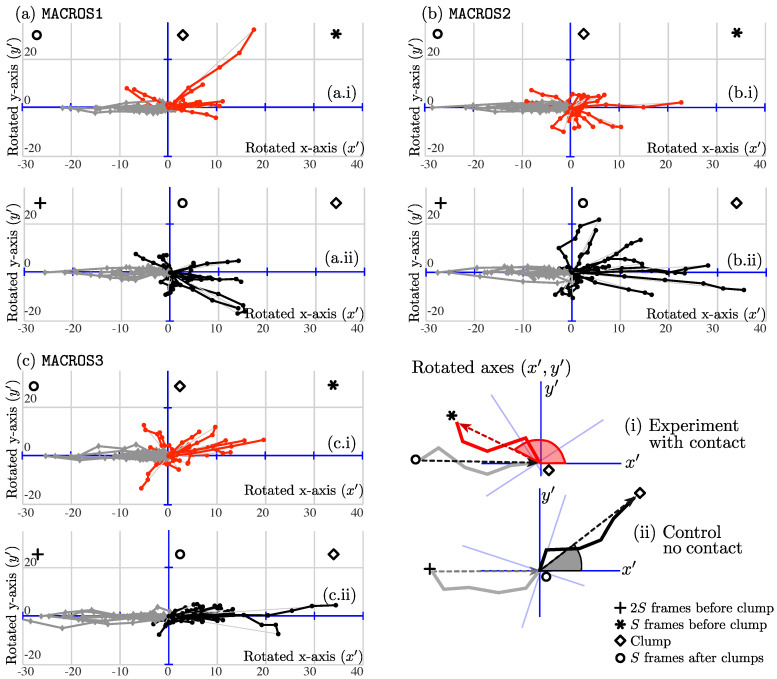
Comparison of the changes in track directions with or without *clump* interaction within the all datasets, (**a**) N=15; (**b**) N=17 and (**c**) N=20. (**c**) Correspond to the results reported in [18]. The sold line represents a cell’s trajectory. Each line can be read from the utmost left point and continuing along the line. For all datasets, (**a**) MACROS1; (**b**) MACROS2 and (**c**) MACROS3, (i) illustrates cells entering or exiting a *clump*, where the origin (⋄) represents the *clump* formation. Grey lines represent S=5 time frame points of each cell’s track before entering a *clump*. Red lines represent 5 time frame points of each cell’s track after exiting a *clump*. (ii) illustrates the movement of cells before entering a *clump*, where the origin (∘) represents a chosen arbitrary point (time frame 6).

All datasets were segmented and tracked. Tracks were selected based on the criteria described in Section 3.4. In total, fifty-two cases were found with N=15,17,20 respectively for each dataset. To represent the tracks and changes in directions for all datasets and compare them to the control tracks, Figure 9 is presented to qualitatively show the hypothesis depicted in Section 3.4 and Figure 1. The figure contains a key taken from the explanatory Figure 8.

Several differences can be observed in the tracks containing cell–cell contact, varying depending on the dataset. To assess the changes of angles of all experiments collected, the mean and median of the angle changes were compared from experiments to control. Referring to Figure 9, Table 3 summarizes the angle comparisons made with the mean and standard deviation in each case. The Wilcoxon Signed Rank test [22] was implemented in all cases, to compare the median of the measurements and a normal *t*-Test was implemented to compare the means.

**Figure 10:**
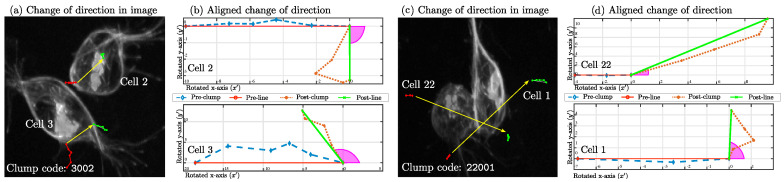
Examples of change of direction before and after a *clump*. Two examples of cells interacting in three different *clumps*: 3002 (**a**,**b**) and 22001 (**c**,**d**). (**a**,**c**) Red line (*-) shows the orientation of movement before the *clump*, and a green line (.-) represents the positions of movement after. A yellow arrow was superimposed on the image to show the trajectory of the cell inside the *clump*. (**b**,**d**) Simplified view of the cells’ changes in orientation. The cells’ path before the *clump* is represented in blue (-·-). The path of the cell after the *clump* is shown in orange (:*). The angle arc of orientation is shown in magenta.

A more thorough exploration of the cases and the tracks was implemented, considering the time in *clump* (TC) parameter. First, consider the average TC per the datasets, where MACROS1 had an average of TC=3.6±3.18, MACROS2 had an average of TC=19.65±24.96 and MACROS3 had an average of TC=12.30±14.25. Visually, TC can be observed in Figure 10, which displays two examples of cells interacting through a given *span* and the orientation lines before (red) and after (green) the *clump*. *Clumps* shown contain the codes 3002 and 22001, which have two very different values of TC, as can be observed from the yellow lines.

**Figure 11:**
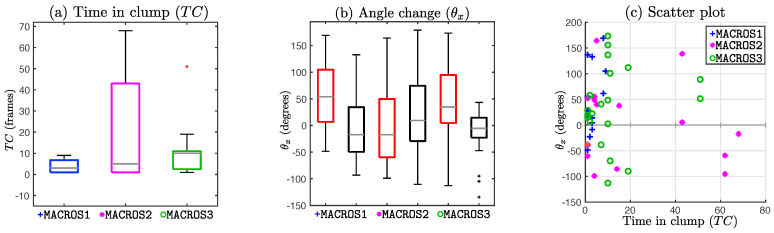
Comparison of the time in *clump* and the angle of variation. (**a**) Boxplot diagram comparing time in *clump* (TC) per dataset; (**b**) Boxplot comparing the value of the measured angle change from the contact (red) and the control (black) experiments; (**c**) Scatter plot of time in *clump* vs. angle change in contact experiments per dataset.

**Figure 12:**
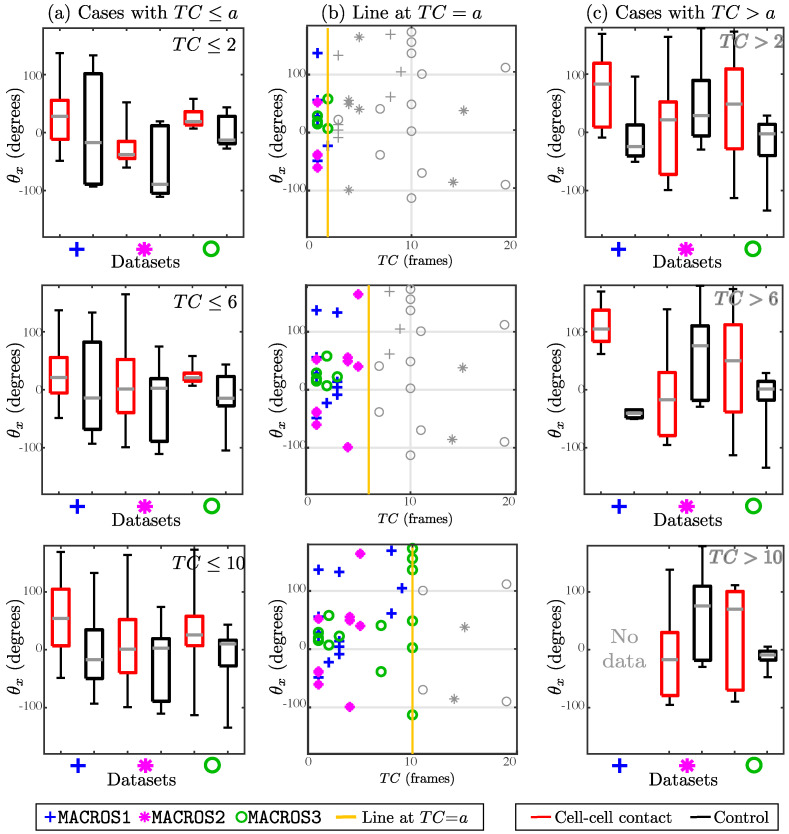
Comparison of angle change, depending on the time spent in *clumps*. In both datasets, boxplots of the angle change were generated for experiments with cell–cell contact (red) and control movement (black). The figure should be read by rows, where each one corresponds to a specific number of frames the cells remained in a *clump* (Time in *clump*: TC=a,a=2,6,10). The left column shows boxplots of the experiments that fulfil the criterion TC≤a and the right column show the experiments where TC>a. The middle column shows scatter plots showing all cases from both datasets.

Figure 11 and Figure 12 explore the differences between change in angle and time in *clump* (TC). From Figure 11a, it can be observed that TC range for datasets MACROS1 and MACROS3 is much smaller compared to the range of the TC for dataset MACROS2. The scatter plot Figure 11c shows a distinct gap in the range 20<TC<40, and most of the cases in the range 0≤TC<10.

In Figure 12, a comparison was made between the angle change depending on TC. Each row represents the comparison of $\theta_{x}$ between cases with cell–cell contact and control cases, when selecting only cases with TC≤a where a=2,6 and 10. Although most cases are broadly similar, cases with TC≤2 and TC≤6 show a more distinctive difference between control and cell–cell contact cases, with control cases (black box plots) showing a greater range in almost all cases. A final experiment was run, in which the value of the angle change $\theta_{x}$ was compared between the control and interaction experiments presenting a TC<10. The cases satisfying TC<10 in all datasets were combined, giving a total of 33, where the mean for interaction cases was $31.65^{\circ }\pm 64.05$ and the control cases was $-8.87^{\circ }\pm 63.38$. The Wilcoxon Signed Rank test produced a *p*-value of 0.03, providing statistical significance to the difference.

## 5. Discussion

Preliminary works in this field have provided separate analyses of macrophages data. First, ref. [15] focusing only in the disambiguation of the lost information of overlapping regions, without including the temporal context. Then, ref. [16] showed an analysis of the shape evolution of cells that do not overlap. Finally, the initial study to understand macrophages’ direction changes as a consequence of cell–cell contact was presented in [18]. In this work, macrosight is presented as a complete framework which includes the routines for data handling, segmentation as well as shape and tracks analysis. In particular, the methodologies described in this work analyze the movement of macrophages exploiting the overlapping of cells observed in the green channel.

This work is an extension to the work shown in [18]. The principal extension made was the presentation of a full framework of routines. The main algorithm provides insights into the relationship between cell–cell contact events, interactions of cells and movement patterns, as shown by Figure 9. The figure shows tracks to be different between the two cases. The cases of cell–cell contact show less movement before and after contact, shown by the smaller lines; a higher variability is observed in control cases. Each dataset also presents unique differences in the comparisons. In MACROS2, for example, the tracks appear more curved after the interaction; and in MACROS3, the ranges of change in direction are more distinct. Even though the differences between contact and control cases are noticeable, the characterization of the movement should not be reduced to the analysis of a single variable, in this case the angle of changed direction. More variables like speed, curvature could be incorporated. From a biological standpoint, the moment cells come in contact could be determined much sooner if the microtubules with lower intensities are also segmented and tracked.

The time in *clump*, TC, was found to be a relevant parameter for the measured angle change for different reasons. Firstly, the value of TC per *clump* appeared to be smaller where statistical significance was achieved, as seen in Table 3. Secondly, from Figure 11 and Figure 12 display the differences in the angle changes and their ranges if the experiments are restricted to keeping a low time in *clump* (TC<10). Finally, as mentioned in Section 2, each frame is taken every 10 s, and the time cells remain in contact should not exceed 1–2 min (TC∈[6,12]) to be considered an interaction relevant to explore. Through the analysis made in this work, the time in *clump* was found to be helpful when assessing the change of direction in values of TC<10. The result was not previously found in [18], therefore it fuels the need for a more thorough analysis of the tracks’ parameters, attempting to characterize them.

The limitations of the algorithm involved the underlying limitations of the segmentation and tracking methods at dealing with complicated interactions. As mentioned in Section 3.4, experiments were chosen manually, by looking at the *clump span* of each case. Upon verification of the tracks, which consists of manually comparing the segmented nuclei and tracks, the dataset MACROS1 presented some inconsistencies such as nuclei changing their track identifier, thus complicating the choosing of experiments. The problems with the tracks could be inherited from the limitations of the keyhole algorithm, which only considers velocity and previous direction to estimate the upcoming position. The reader is referred to Figure 9b.i, where the red tracks appear clearly curved.

The problems could also be due to segmentation, as MACROS1 contains more variability in the intensities than MACROS2 and MACROS3, as well as a larger number of cells interacting in each *clump*. Future work could improve this by incorporating post-processing to the tracks.

## 6. Conclusions

This work presents a novel system for the analysis of movement of macrophages and other objects. The main contribution is the integration image analysis techniques into a robust framework to perform automated and semi-automated (such as the selection of the experiments) analyses of movement objects. In particular, this study introduced a methodology for the analysis of movement of macrophages, and the relationship between cell–cell contact and changes in the trajectories of the participating cells.

Despite some encouraging results, the differences shown by macrosight should not be interpreted as conclusive, but as encouraging insights into future research. Some future developments proposed include the following:

(i) A deeper understanding is needed in terms of the anticipation before recording an experiment, the number of frames to be taken before and after the start and end of the *clump*, *S*. As suggested by Stramer et al. [8], cells appear to elongate before an interaction, thus such elongation could aid in determining the value of *S* automatically. Macrosight already contains the functionality to measure the elongation of the cells, as reported in [16].
(ii) the *time in clump*, TC, was introduced, but not studied thoroughly. *Clumps* could introduce other variables not accounted for, or interactions not quantified
(iii) Cells involved in *clumps* where more than two cells come in contact were not considered, to control the variability of the experiments. However, such inclusion would allow several more cases of study, for instance, dataset MACROS1 contained over 40 cases including the 15 presented in this work.
(iv) More importantly, the calculation of the direction was done simply by taking two points before and after the *clump*, and only one variable (change of direction) was measured. However, as mentioned before, the tracks have more complex parameters, such as speed, curvature, or acceleration. A thorough characterization of the tracks’ ontological properties, as mentioned in [13]; could allow for the inclusion of several tracks in the analysis, aiding in a conclusive determination of cell movement changes influenced by contact.

Samples of the data and the code of the macrosight framework are available at https://github.com/alonsoJASL/macrosight under a GNU 3 open source license, or upon request to the authors.

## Figures and Tables

**Table 1:** Brief description of the main parameters measured per track.

Field	Description
Time frame	Frame in the dataset where the following parameters were measured.
Position $\left(x_{t},y_{t}\right)$	Cartesian coordinates of the centroid of the detected nucleus.
Velocity	Calculated with the position of the previous frame.
Track label	Unique identifier for each track.

**Table 2:** Examples of *clump* codes created through the track labels obtained by PhagoSight by applying the defined codes. See text for detailed explanation.

*Clump* Code	Code Construction c(·)	Tracks Within *Clump*
2001	c(2,1)=2000+1	2,1
3002	c(3,2)=3000+2	3,2
5003002	c(5,3,2) = 5,000,000 + 2000 + 1	5,3,2

**Table 3:** Angle change $\left(\theta_{x}\right)$ comparison per dataset. The mean and standard deviation angle change were calculated, and the results of the statistical tests comparing both contact and control experiments are shown. In moments where the null hypothesis could not be rejected are highlighted in red.

DATASET	Cell–Cell Contact	Control	WILLCOXON	*t*-Test
	Mean (std)	Mean (std)	*p*-Value	*p*-Value
MACROS1	53.79 (64.25)	−4.34 (74.18)	0.08	0.03
MACROS2	0.61 (77.31)	15.48 (78.10)	>0.05	>0.05
MACROS3	37.40 (77.65)	−15.59 (46.68)	0.02	0.01
ALL	30.10 (75.70)	−2.19 (66.42)	0.03	0.02

## Abbreviations

The following abbreviations are used in this manuscript:

ΔDirection	Change of direction
GFP	Green Fluorescent Probe
MIUA	Medical Image Understanding and Analysis
$T_{i}$	Track acquired with identifier *i*
TC	Time in *clump*
